# Staged versus One-Time Complete Revascularization with Percutaneous Coronary Intervention in STEMI Patients with Multivessel Disease: A Systematic Review and Meta-Analysis

**DOI:** 10.1371/journal.pone.0169406

**Published:** 2017-01-20

**Authors:** Zhenwei Li, Yijiang Zhou, Qingqing Xu, Xiaomin Chen

**Affiliations:** 1 Department of Cardiology, The Affiliated Hospital Ningbo No.1 Hospital, Zhejiang University, Ningbo, PR China; 2 Department of Cardiology, The First Affiliated Hospital, School of Medicine, Zhejiang University, Ningbo, PR China; 3 Department of Nephrology, The Affiliated Hospital Ningbo No.1 Hospital, Zhejiang University, Ningbo, PR China; Azienda Ospedaliero Universitaria Careggi, ITALY

## Abstract

**Introduction:**

In patients with acute ST-elevation myocardial infarction (STEMI), the preferred intervention is percutaneous coronary intervention (PCI).Whether staged PCI (S-PCI) or one-time complete PCI (MV-PCI) is more beneficial and safer in terms of treating the non-culprit vessel during the primary PCI procedure is unclear. We performed a meta-analysis of all randomized and non-randomized controlled trials comparing S-PCI with MV-PCI in patients with acute STEMI and MVD.

**Methods:**

Studies of STEMI with multivessel disease receiving primary PCI were searched in PUBMED, EMBASE and The Cochrane Register of Controlled Trials from January 2004 to December 2014. The primary end points were long-term rates of major adverse cardiovascular events and their components—mortality, reinfarction, and target-vessel revascularization. Data were combined using a fixed-effects model.

**Results:**

Of 507 citations, 10 studies (4 randomized, 6 nonrandomized; 820 patients, 562 staged PCI and 347 one-time, complete multi-vessel PCI) were included. S-PCI compared to MV-PCI significantly reduced mortality both long-term (OR 0.44, 95% CI 0.29–0.66, P<0.0001, I^2^ = 0%) and short-term (OR 0.23, 95% CI 0.1–0.51, P = 0.0003, I^2^ = 0%). There was a trend toward reduced risk of MACE with s-PCI compared with MV-PCI (OR 0.83, 0.62–1.12, P = 0.22, I2 = 0%). No difference between S-PCI and MV-PCI was observed in reinfarction (OR 0.97, 0.61–1.55, P = 0.91, I^2^ = 0%), or target vessel revascularization (OR1.17, 95% CI 0.81–1.69, P = 0.40, I^2^ = 8%).

**Conclusions:**

The staged strategy for non-culprit lesions improved short- and long-term survival and should remain the standard approach to primary PCI in patients with STEMI; one-time complete multivessel PCI may be associated with greater mortality risk. However, additional large, randomized trials are required to confirm the optimal timing of a staged procedure on the non-culprit vessel in STEMI.

## Introduction

Percutaneous coronary intervention (PCI) has become the preferred reperfusion strategy in patients with acute ST-elevation MI (STEMI) compared to intravenous thrombolytic therapy [1–3]. Approximately 40–50% of patients with STEMI have at least 1 additional severe stenosis lesion of >50% in the non-culprit vessel [4,5]. Patients with multivessel disease (MVD) have worse clinical outcomes in terms of major adverse cardiovascular events (mortality, reinfarction, and target-vessel revascularization) than patients with single-vessel disease [4,6,7]. One-time, multivessel PCI may contribute to a higher risk of complications such as stent thrombosis [8–10], inflammatory burden [11], and contrast-induced nephropathy [12] associated with STEMI. Previous guidelines recommended that patients presenting with STEMI without hemodynamic instability undergo PCI of the culprit vessel (CV-PCI) rather than PCI of the non-infarct related artery (IRA) [13,14]. However, recent advancements in interventional cardiology to reduce procedure time, the improvement in strategies to reduce the risk of acute kidney injury, the widespread use of new types of drug-eluting stents, and novel antiplatelet therapy to reduce the risk of stent thrombosis have all made multi-vessel PCI more reliable, predictable, and reproducible [15,16]. Performing one-time, multi-vessel PCI at the time of primary PCI may be safe and beneficial in patients with STEMI [17–20]. The PRAMI (Preventive Angioplasty in Acute Myocardial Infarction) trial and CvLPRIT (Complete versus Lesion-only Primary PCI) trial demonstrated that MV-PCI significantly reduced adverse cardiovascular events compared to CV-PCI [18,19]. Multivessel-completed PCI guided by FFR (fractional flow reserve) significantly reduced the risk of further events (such as repeat revascularization, all-cause death, and nonfatal MI) compared to culprit artery–only PCI in the DANAMI 3 PRIMULTI (Third Danish Study of Optimal Acute Treatment of Patients with ST-segment Elevation Myocardial Infarction) trial [17]. No differences in all-cause death, nonfatal MI, and stroke in staged PCI or culprit-only PCI were observed in the PRAGUE-13 (Primary Angioplasty in Patients Transferred From General Community Hospitals to Specialized PTCA Units With or Without Emergency Thrombolysis) trial [20]. Based on these findings, the American College of Cardiology (ACC)/American Heart Association (AHA)/Society for Cardiovascular Angiography and Interventions (SCAI) currently recommend consideration of multivessel PCI either at the time of primary PCI or as a staged procedure(Class IIb), modified from culprit vessel PCI (CV-PCI) in the absence of hemodynamic instability (Class III)[21]. However, it is unknown whether staged PCI (S-PCI) or one-time complete PCI (MV-PCI) is the safer and more beneficial procedure to treat the non-culprit vessel during the primary PCI procedure. Therefore, we conducted a meta-analysis of all randomized controlled trials (RCTs) and non-randomized controlled trials (non-RCTs) to compare the cure effects of S-PCI and MV-PVI in STEMI patients with multivessel disease.

## Methods

### Study selection and search criteria

**Fig 1:**
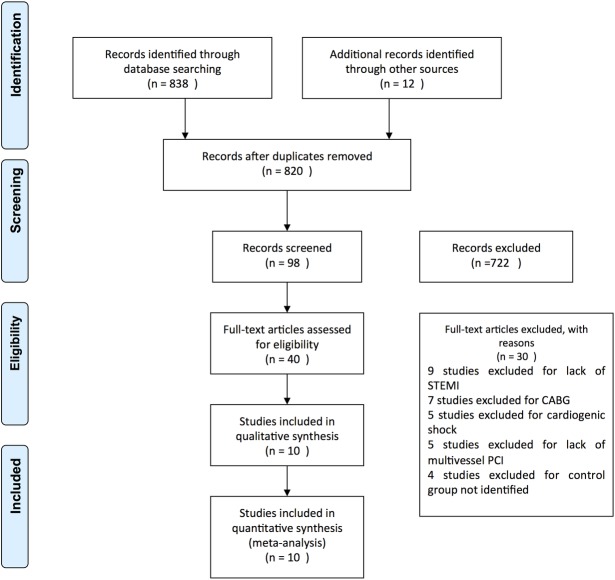
PRISMA Flow diagram of included studies.

This meta-analysis was performed in accordance with the Cochrane Handbook for Systematic Reviews and Interventions [22] and was reported following the PRISMA statement [23]. Two authors (Zhenwei Li & Yijiang Zhou) independently searched PubMed, the Cochrane Central Register of Controlled Trials and EMBASE in English-language publications from January 2004 to December 2014. The following keywords and medical subject headings (MeSH) were used: ““coronary angioplasty,” “ST-elevation myocardial infarction,” “percutaneous coronary intervention,” “multivessel PCI,” “staged PCI,” “complete revascularization,” “non-culprit,” and “myocardial infarction.” Both RCTs and non-RCTs comparing staged vs multivessel PCI in patients with STEMI and MVD undergoing primary PCI without hemodynamic instability were included. We screened the abstracts (i.e., unpublished citations) and full-text citations for eligibility in the meta-analysis. To eliminate negative publication bias, unpublished citations were also included, and the relevant references were collected through a manual search. The PRISMA flow diagram for study selection is presented in Fig 1. A full electronic search strategy (no limits) performed in PUBMED can be reviewed in the Table 1.

**Table 1:** Search strategy used for PUBMED.

**1**	"multivessel"
**2**	"staged"
**3**	"multi-vessel"
**4**	**1 or 2 or 3**
**5**	"stent"
**6**	"dilatat*"
**7**	"balloon"
**8**	stents[MeSH Terms]
**9**	balloon dilation, coronary artery[MeSH Terms]
**10**	5 or 6 or 7 or 8 or 9
**11**	"myocard* infarct*"
**12**	myocardial infarction[MeSH Terms]
**13**	11 or 12
**14**	"percutaneous coronary intervention,"
**15**	"angioplasty"
**16**	angioplasty, transluminal, percutaneous coronary[MeSH Terms]
**17**	14 or 15 or 16
**18**	4 and 10 and13 and17

((((("multivessel") OR "staged" OR "multi-vessel") AND (((("stent" OR "dilatat*" OR "balloon") OR stents[MeSH Terms]) OR balloon dilation, coronary artery[MeSH Terms])) AND (("myocard* infarct*") OR myocardial infarction[MeSH Terms])) AND (((("percutaneous coronary intervention,") OR "angioplasty")) OR (angioplasty, transluminal, percutaneous coronary[MeSH Terms]))

### Methodological quality assessment and data abstraction

The Cochrane Collaboration tool was used to assess the quality of the abstracted studies to assess the risk of bias [22] in the RCTs. The Newcastle Ottawa Scale [24] was used to evaluate the quality of the non-RCTs. A star was assigned to 3 aspects of the study: selection (4 criteria), outcome (3 criteria) and comparability (1 criterion). A study could have up to 1 star for each criterion, and thus 8 stars indicate excellent quality, whereas no stars indicate poorest quality.

Data were extracted systematically from the intervention (S-PCI) and control (MV-PCI). The primary end points were long-term rates of major adverse cardiovascular events and their components—mortality, reinfarction, and target-vessel revascularization. The secondary end point was short-term mortality. A fixed-effects model was used in case of low heterogeneity.

### Data analysis

All statistical analyses were performed using Review Manager (RevMan 5.2, Cochrane Collaboration, Nordic Cochrane Center, Copenhagen, Denmark). Odds ratios (ORs) with 95% CIs were used as summary estimates. Given the low event rates and small size of selected studies, the Mantel-Haenszel method was used to calculate the pooled OR with the fixed-effects model. Study heterogeneity was measured using the I^2^ index and Cochran’s Q, where an I^2^ greater than 60 and P<0.1 represent severe heterogeneity. Sensitivity analyses were performed to explore heterogeneity. Depending on the study design, a subgroup of the RCTs and non-RCTs was generated for each outcome to help explain heterogeneity. A “funnel plot” approach was used to avoid the potential for publication bias.

## Results

### Search and selection of studies

**Fig 2:**
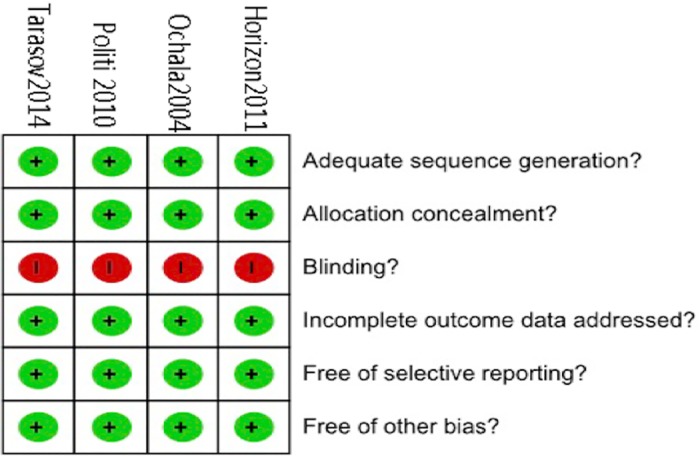
The Cochrane Collaboration Tool was used to estimate the risk of bias for each included randomized study.

**Fig 3:**
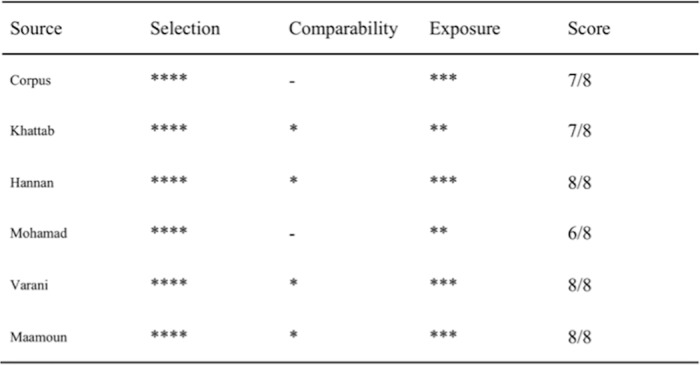
The Newcastle Ottawa Scale was used to estimate the risk of bias for each included non-randomized study.

As shown in Fig 1, 850 abstracts were retrieved, and 40 were selected. Of these 40 eligible full-text studies, 30 studies were excluded due to lack of STEMI (n = 9), lack of multivessel revascularization (n = 5), failure to identify the control group (n = 4), cardiogenic shock (n = 5), and inclusion of coronary artery bypass grafting surgery (n = 7). Ten studies fulfilled the eligibility criteria and were included in the present systematic review. Of the 10 studies (4 RCTs [25–28] and 6 non-RCTs [10,29–33]), 820 patients were included (562 S-PCI and 347 MV-PCI). Table 2 presents the characteristics of the included studies. The mean long-term follow-up was 14 months. The quality calculation for the RCTs and non-RCTs are presented in Fig 2 and Fig 3.

**Table 2:** Characteristics of the 10 included studies.

Author and year	Study design	No. of patients	Comparison arms	Inclusion criteria	exclusion criteria	Outcomes
Politi et al, 2010 [25]	Randomized	214	MV-PCI, CV-PCI and S-PCI	STEMI with ≥70% stenosis of ≥2 coronary arteries or major branches	Cardiogenic shock, LM disease, pervious CABG, severe valvular heart disease or unsuccessful procedure	In-hospital mortality; long-term mortality, cardiac death, MI, repeat revascularization, rehospitalization, CABG, PCI, MACE
Horizon et al, 2011 [34]	Randomized	668	MV-PCI and S-PCI	STEMI with MVD and 1–3 lesions in non-culprit artery technically amenable to revascularization by stent	Lesion in vein and arterial grafts, prior angioplasty, thrombolytic, cardiogenic shock, platelet count <100,000 cells/mm^3^ or hemoglobin <10 g/dl	1-y MACE
Ochala et al, 2004 [27]	Randomized	92	MV-PCI and S-PCI	At least 1 significant (≥70%) stenosis eligible for PCI in a coronary artery other than the IRA	Left main, cardiogenic shock, target lesion in non-IRA, not suitable for PCI (diffuse, diameter <2.5), high tortuosity, lesion within orifices of large side branch, renal insufficiency or presence of single kidney, contraindication to antiplatelet therapy, previous CABG, valvular heart disease requiring surgery, pregnancy	LVEF, all causes of death, AMI, urgent revascularization (including TVR), major and minor bleeding complications, worsening of the CCS class, unstable angina, cardiovascular hospitalization
Tarasov, 2014 [28]	Randomized	89	MV-PCI and S-PCI	PCI using a zotarolimus-eluting stent; subject must have significant stenoses (≥70%) of two or more of the coronary arteries and require primary PCI for acute ST elevation myocardial infarction (STEMI) within 12 h.	Single lesions; acute heart failure Killip III-IV; ≥50% left main stenosis; Small vessels diameter (<2.5 mm); known hypersensitivity or contraindication to any of the following medications: heparin, aspirin, both clopidogrel and ticlopidine, zotarolimus	6 month MACE
Corpus et al [29]	Non-randomized	506	Culprit PCI vs culprit PCI + multivessel PCI during the index catheterization or staged during the index hospitalization	STEMI with ≥70% stenosis of ≥2 epicardial arteries	PCI of vain graft or after angioplasty, LM, planned staged revascularization	In-hospital mortality; 30-d mortality, reinfarction, TVR, CABG, MACE; 1-y mortality, reinfarction, TVR, CABG, MACE
Khattab et al [10]	Non-randomized	70	Culprit PCI (with possible 70 staged or ischemia-driven PCI of non-culprit lesions) vs culprit PCI +multivessel PCI during the index catheterization	STEMI with ≥70% stenosis of ≥2 coronary arteries or major branches	Non-IRA diameter <2.5 mm, LM disease, previous MI	30-d mortality, MI, TVR, stent thrombosis, CVA, bleeding, MACE; 1-y mortality, MI, TVR, non-TVR, total revascularizations, MACE
Hannan et al [31]	Non-randomized	1434	Culprit PCI vs culprit PCI + multivessel PCI during index catheterization staged PCI during index admission or staged PCI within 60 d	STEMI with MVD	LM disease, prior thrombolysis, prior CABG, cardiogenic shock, missing EF	In-hospital mortality; 12-mo mortality; 24-mo mortality; 42-mo mortality
Mohamad et al [32]	Non-randomized	63	Culprit PCI vs culprit PCI + multivessel PCI during index hospitalization or at a later date	STEMI with ≥70% stenosis of ≥2 coronary arteries	Single-vessel disease, unable to undergo coronary angiography within 3 h of hospital presentation, ≥12-h symptom presentation	1-y mortality, MACE
Varani et al [33]	Non-randomized	399	Culprit PCI vs culprit PCI + multivessel PCI during index catheterization or staged within 24 h or predischarge	STEMI with N70% stenosis of ≥2 epicardial arteries or major branches	Occlusion after prior angioplasty, cardiogenic shock, pulmonary edema	In-hospital mortality, PCI, major vascular complications; 30-d mortality; long-term (630±366 d) mortality
Maamoun et al [30]	Non-randomized	19	MV-PCI and S-PCI	STEMI with ≥70% stenosis of ≥2 epicardial arteries or major branches	Patients with cardiogenic shock, pulmonary edema, and left main coronary artery disease	1-y mortality, MACE

### Long-term major adverse cardiovascular events

**Fig 4:**
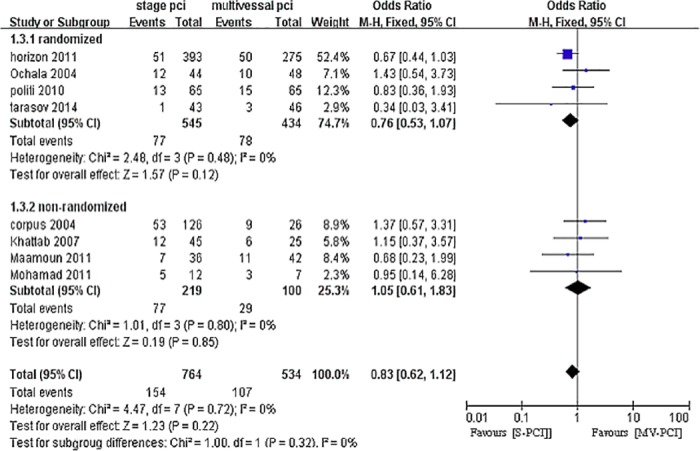
Forest plot of long-term MACE.

Overall, seven studies reported long-term MACE [10,25,27–30,32,34]. There was a trend toward reduced risk of MACE with S-PCI compared with MV-PCI with no heterogeneity, although the trend did not reach statistical significance (OR 0.83, 95% CI 0.62–1.12, P = 0.22, I^2^ = 0%), as shown in Fig 4.

### Short-term mortality

**Fig 5:**
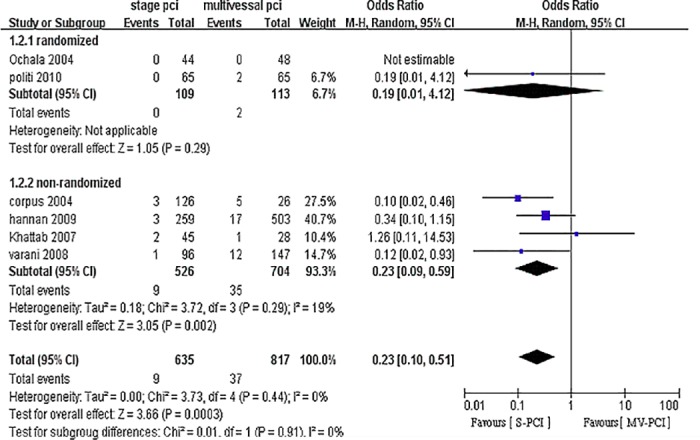
Forest plot of short-term mortality.

Six studies reported in-hospital death [25,27,29,31] [10,15] (Fig 5). Mortality was significantly lower for S-PCI compared with MV-PCI. Improved in-hospital survival was observed for S-PCI, with no heterogeneity (OR 0.23, 95% CI 0.1–0.51, P = 0.0003, I^2^ = 0%).

### Long-term mortality

**Fig 6:**
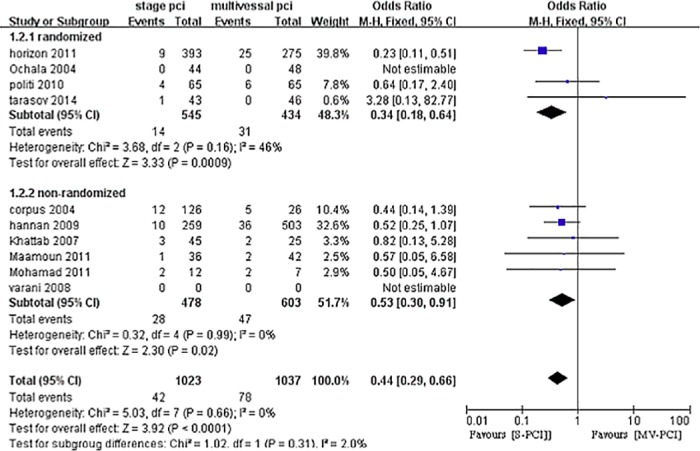
Forest plot of long-term mortality.

Nine studies reported long-term mortality (4 RCTs [25,27,28,34] and 6 non-RCTs) [10,29–33] (Fig 6). The mean follow-up time was 14 months. The combined analysis indicated a survival benefit for S-PCI compared with MV-PCI (OR 0.44, 95% CI 0.29–0.66, P<0.0001, I^2^ = 0%).

### Long-term repeat myocardial infarction

**Fig 7:**
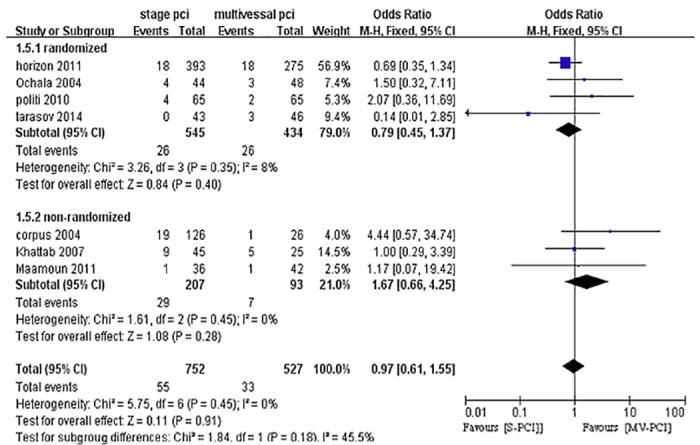
Forest plot of long-term Re-mi.

Six studies reported long-term repeat myocardial infarction (re-MI) after long-term follow-up [10,25,27,29,32,34]. There was no significant difference between S-PCI and MV-PCI. S-PCI had no effect on re-MI, with no heterogeneity (OR 0.97, 95% CI 0.61–1.55, P = 0.91, I^2^ = 0%), as shown in Fig 7.

### Long-term target vessel revascularization (TVR)

**Fig 8:**
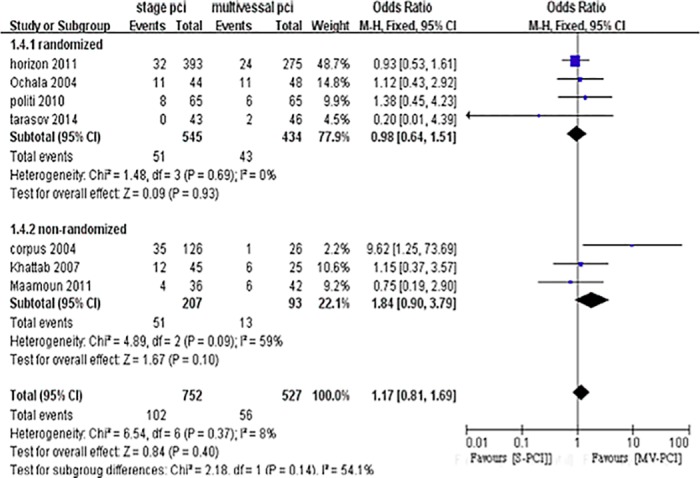
Forest plot of long-term TVR.

Comparing S-PCI with MV-PCI revealed no significant difference in target revascularization in the RCTs [25,27,34], with no heterogeneity (OR 0.98, 95% CI 0.64–1.51, P = 0.09, I^2^ = 0%) (Fig 8). In the non-RCTs [10,29,30], enhanced revascularization was observed for MV-PCI, with moderate heterogeneity (OR 1.84, 95% CI 0.90–3.79, P = 0.10, I^2^ = 59%), mainly driven by the results of the non-RCT performed by Corpus [29].

### Study quality and publication bias

Both RCTs and non-RCTs were included in our meta-analysis. The high quality of the non-RCTs was indicated by the Newcastle Ottawa Scale score of ≥6/8. The Cochrane Collaboration tool for assessing risk of bias also confirmed the high quality of the RCTs. The funnel plot revealed no publication bias.

## Discussion

The present meta-analysis of randomized and observational studies is one of the largest to support staged multivessel PCI as feasible and safe in the context of STEMI. The main findings are that, compared with MV-PCI, S-PCI is (1) associated with a trend toward reduced risk of the composite primary endpoint of MACE odds and (2) associated with lower short-term and long-term mortality odds.

Primary PCI and PCI for STEMI patients were associated with lower subsequent revascularization rates and lower mortality, as demonstrated by RCTs and observational studies [35–38]. However, multivessel disease in STEMI patients continues to pose a major challenge and has been associated with substantially worse prognosis compared with single-vessel disease [4,6,7]. Earlier clinical practice guidelines recommend that in STEMI patients, only the culprit vessel be initially treated, unless hemodynamic compromise is present [13,14]. These recommendations are based on data from observational studies [29,31] rather than RCTs. Whether this remains the appropriate course of action is unclear. Continuous refinements in interventional techniques coupled with advancements in drug-eluting stents and antithrombotic strategies have led to significant improvements in procedural success and long-term clinical outcomes [39]. Multivessel PCI may have advantages over culprit-only PCI because instable plaque may emerge in not only the infarct-related artery but also the non-infarcted coronary vasculature [40]. In addition, stable coronary artery disease is associated with improved long-term clinical prognosis when performing the complete revascularization strategy [41–43]. Therefore, some cardiologists perform immediate multivessel PCI in spite of the guidelines. Currently, four RCTs suggest that multivessel PCI may be more safe and beneficial in STEMI patients with multivessel disease [17–20]. Based on the Data Supplement, the current ACC/AHA guidelines have been modified so that revascularization of a noninfarct artery may be considered for select STEMI patients with multivessel disease who are hemodynamically stable, whether in a later-stage procedure or at the time of primary PCI (class of recommendation II; level of evidence B) [21]. However, most of these RCTs made comparisons between complete PCI and culprit lesion-only primary PCI, and thus it is unknown if S-PCI or MV-PCI is the more beneficial and safer procedure to treat the non-culprit vessel. Kowalewski et al performed a comprehensive analysis that found that MV-PCI was associated with a significant 41% reduction of MACE (death, recurrent myocardial infarction and repeat revascularization) compared with non-complete MV-PCI. However, significant controversy remains. Bainey’s meta-analysis indicated that staged PCI compared to culprit lesion-only PCI improved short- and long-term survival for lower rates of repeat PCI. However, once multivessel PCI was performed during index catheterization, hospital mortality increased [44]. Vlaar et al performed a smaller pairwise and network meta-analysis that demonstrated that a staged approach in STEMI with multivessel disease had lower short-term and long-term mortality than culprit PCI and MV-PCI [45]. We confirmed these findings and further supported long-term survival (~14 months) with staged PCI. Improved in-hospital and long-term survival were observed in a staged fashion; however, when multivessel PCI was performed during index catheterization, increased mortality was observed. Given the lack of preference for staged PCI in the current guidelines, these findings are particularly important. Performing one-time complete PCI may contribute to the acute phase of STEMI, in which the pro-thrombotic and inflammatory environment may lead to acute stent thrombosis. Moreover, the physiological state and tenuous hemodynamics often result in the complication of acute left ventricular dysfunction. In addition, when PCI is performed in the acute phase [10,34]. The higher amount of contrast used during primary PCI contributed to contrast-induced nephropathy [46]. Alternatively, a stable environment will be supported when performed in a staged procedure. In addition, the surgeon always overestimates the severity of non-culprit lesions in the STEMI setting, mainly due to diffuse coronary artery spasms or endothelial dysfunction [47]. The result may be unnecessary intervention and an increase in procedural risk without the added benefit.

The present meta-analysis demonstrates that SV-PCI is superior to MV-PCI for reducing mortality in STEMI patients with multivessel disease without hemodynamic instability. However, the optimal timing of a staged procedure on the non-culprit vessel after culprit PCI has been performed remains unclear. An electronically distributed survey conducted by the market research department of the American College of Cardiology revealed that although the majority of interventional cardiologists agreed on staging the non-culprit vessel PCI at a later date, there was significant variability of opinions regarding the timing of staged PCI. Only 22% of the respondents would perform non-culprit vessel PCI during the same hospitalization; the majority of cardiologists (64%) recommended a timeframe of >15 days for the second PCI after initial revascularization [48]. Data from New York State’s PCI registry indicated that patients who underwent staged non-culprit vessel PCI within 60 days after the index procedure had lower short- and long-term mortality rates compared to culprit vessel PCI. However, these survival benefits were not observed in staged non-culprit vessel PCI during the index hospitalization [31]. A sub-study included in our meta-analysis, the Harmonizing Outcomes With Revascularization and Stents in Acute Myocardial Infarction (HORIZONS-AMI) trial, observed improved 1-year outcomes in STEMI patients with multivessel disease undergoing staged PCI (median 30 days) compared with one-time complete PCI [34]. There are theoretical disadvantages of an early second intervention for the non-culprit vessel within the same hospitalization. The myocardial injury sustained from the acute STEMI coupled with a pro-thrombotic and inflammatory state may increase procedural risks [11,49]. The accumulation of additional contrast load in short intervals may increase the risk of developing nephropathy [46,50]. Non-infarcted myocardium may be jeopardized if procedural complications arise in the non-culprit vessel and the infarcted myocardium has not recovered for a sufficient amount of time. In addition, strong antithrombotic and anticoagulation therapy may lead to a higher incidence of access-site bleeding and vascular complications if the double puncture is executed in the same hospitalization. These risks may be reduced once sufficient recovery time is provided to perform the staged procedure. However, once coronary plaques stabilize from the index event, the multiple unstable coronary plaques (demonstrated by the angiographic and angioscopic studies) will be removed from the culprit lesion, prompting a staged in-hospital PCI [40,51,52]. In a single-center, retrospective cohort study of STEMI patients with multivessel disease, non-culprit vessel PCI performed <24 hours after primary PCI and staged PCI before hospital discharge achieved a similarly low 30-day mortality (2.1%), in contrast to multi-vessel PCI performed during primary PCI (9.9%) [33]. Joshua [53] supports the safety and feasibility of staged PCI within the same hospitalization (mean interval of 3 days from index to staged PCI) as primary PCI, which achieved similar procedural success and in-hospital outcomes as staged PCI at a separate hospitalization (mean interval of 29.5 days from index to staged PCI). Further investigation is still required to determine the optimal timing of a staged procedure in STEMI with multivessel disease. However, in China, reimbursement issues may drive the decision for revascularization. Physicians in China may not be compensated for staged PCI performed within 30 days of STEMI PCI and thus may not have the incentive to treat other vessels while the patient is still in the hospital. Therefore, economic concerns drive most healthcare systems to favor the strategy of two procedures performed in separate hospitalizations.

## Limitation

In our meta-analysis, we were forced to include observational non-randomized studies due to a lack of randomized data. We performed randomized vs nonrandomized stratified analyses for the pooled estimate. However, many selection biases and confounding factors remained in the observational studies, even after statistical adjustment. Unpublished abstracts were also included to reduce publication bias. The data of the original included studies were limited to analysis at the trial level rather than the patient level. Therefore, we could not adjust the baseline characteristics of the included patients and multivariate factors; the follow-up and admission medications were also not captured. Moreover, the impact of chronic total occlusions was not fully evaluated due to the absence of reports in most selected studies. A staged and planned strategy for non-culprit vessel PCI may be preferable for these patients given the risk and difficulties in attempting a chronic total occlusion. In addition, when patients wait for staged or planned PCI, clinical events may occur; these events were not adequately described in the included studies. In addition, The exact mechanisms linking staged PCI with better short and long mortality can not be elucidated by the provided data since non-fatal reinfarcion rates were similar, the specific causes of deaths are unknown. Ochala’ study even said MV-PCI in patients with STEMI and MVD leads to quicker and more substantial improvement of LVEF in comparison to S-PCI, However, Horizon’ study said stent thrombosis and bleeding(Major or minor) complications rates were significantly increased in MV-PCI versus S-PCI groups. Maybe the less such complications made staged PCI with better short and long mortality. Finally, survival selection bias in staged PCI patients can potentially affect long-term survival. Therefore, we excluded patients in cardiogenic shock and performed a sensitivity analysis of all studies, which confirmed the survival benefit.

## Conclusion

Our meta-analysis provides new insights on the efficacy and safety of staged PCI compared with one-time complete PCI in patients with STEMI. We observed reduced short- and long-term mortality with a strategy of staged PCI. The results of our study suggest that PCI of the non-culprit vessel should be staged and that many factors and conditions influence the decision of when to stage PCI. However, our findings require additional large-scale, multicenter, randomized controlled studies for confirmation.

## Supporting Information

S1 Table PRISMA checklist. (DOC)

S1 File Original data of the study. (DOCX)

## References

[1] Keeley E, Boura J, Grines C. Primary angioplasty versus intravenous thrombolytic therapy for acute myocardial infarction: a quantitative review of 23 randomised trials. Lancet 2003;361: 13–20. 10.1016/S0140-6736(03)12113-7 12517460

[2] Andersen HR, Nielsen TT, Rasmussen K, Thuesen L, Kelbaek H, Thayssen P, et al A comparison of coronary angioplasty with fibrinolytic therapy in acute myocardial infarction. N Engl J Med. 2003;349: 733–742. 10.1056/NEJMoa025142 12930925

[3] Widimsky P, Budesinsky T, Vorac D, Groch L, Zelízko M, Aschermann M, et al Long distance transport for primary angioplasty vs immediate thrombolysis in acute myocardial infarction. Final results of the randomized national multicentre trial—PRAGUE-2. Eur Heart J. 2003;24: 94–104. 12559941 10.1016/s0195-668x(02)00468-2

[4] Sorajja P, Gersh B, Cox D, McLaughlin M, Zimetbaum P, Costantini C, et al Impact of multivessel disease on reperfusion success and clinical outcomes in patients undergoing primary percutaneous coronary intervention for acute myocardial infarction. Eur Heart J 2007;28: 1709–1716. 10.1093/eurheartj/ehm184 17556348

[5] Dziewierz A, Siudak Z, Rakowski T, Zasada W, Dubiel J, Dudek D. Impact of multivessel coronary artery disease and noninfarct-related artery revascularization on outcome of patients with ST-elevation myocardial infarction transferred for primary percutaneous coronary intervention (from the EUROTRANSFER Registry). Am J Cardiol 2010;106: 342–347. 10.1016/j.amjcard.2010.03.029 20643243

[6] Muller DW, Topol EJ, Ellis SG, Sigmon KN, Lee K, Califf RM. Multivessel coronary artery disease: a key predictor of short-term prognosis after reperfusion therapy for acute myocardial infarction. Thrombolysis and Angioplasty in Myocardial Infarction (TAMI) Study Group. Am Heart J. 1991;121: 1042–1049. 1901190 10.1016/0002-8703(91)90661-z

[7] Chen H, Tsai T, Fang H, Sun C, Lin Y, Leu S, et al Benefit of revascularization in non-infarct-related artery in multivessel disease patients with ST-segment elevation myocardial infarction undergoing primary percutaneous coronary intervention. Int Heart J 2010;51: 319–324. 20966603 10.1536/ihj.51.319

[8] Chan M, Andreotti F, Becker R. Hypercoagulable states in cardiovascular disease. Circulation. 2008;118: 2286–2297. 10.1161/CIRCULATIONAHA.108.778837 19029477

[9] Kereiakes D, Gurbel P. Peri-procedural platelet function and platelet inhibition in percutaneous coronary intervention JACC Cardiovasc Interv. 2008;1: 111–121. 10.1016/j.jcin.2008.01.005 19463287

[10] Khattab A, Abdel-Wahab M, Röther C, Liska B, Toelg R, Kassner G, et al Multi-vessel stenting during primary percutaneous coronary intervention for acute myocardial infarction. A single-center experience Clin Res Cardiol. 2008;97: 32–38. 10.1007/s00392-007-0570-4 17694377

[11] Ohashi Y, Kawashima S, Mori T, Terashima M, Ichikawa S, Ejiri J, et al Soluble CD40 ligand and interleukin-6 in the coronary circulation after acute myocardial infarction. Int J Cardiol. 2006;;112: 52–58. 10.1016/j.ijcard.2005.09.051 16376442

[12] Assali A, Brosh D, Ben-Dor I, Solodky A, Fuchs S, Teplitsky I, et al The impact of renal insufficiency on patients outcomes in emergent angioplasty for acute myocardial infarction. Cathet Cardiovasc Interv. 2007;69: 395–400. 10.1002/ccd.20939 17195964

[13] O'Gara P, Kushner F, Ascheim D, Casey D Jr, Chung M, de Lemos J, et al 2013 ACCF/AHA guideline for the management of ST-elevation myocardial infarction: executive summary: a report of the American College of Cardiology Foundation/ American Heart Association Task Force on Practice Guidelines: developed in collaboration with the American College of Emergency Physicians and Society for Cardiovascular Angiography and Interventions. Catheter Cardiovasc Interv 2013;82: E1–27. 10.1002/ccd.24776 23299937

[14] Task Force on the management of ST-segment elevation acute myocardial infarction of the European Society of Cardiology (ESC), Steg P, James S, Atar D, Badano L, Blömstrom-Lundqvist C, et al ESC Guidelines for the management of acute myocardial infarction in patients presenting with ST-segment elevation. Eur Heart J 2012;33: 2569–2619. 10.1093/eurheartj/ehs215 22922416

[15] Varani E, Guastaroba P, Di Tanna G, Saia F, Balducelli M, Campo G, et al Long-term clinical outcomes and cost- effectiveness analysis in multivessel percutaneous coronary interventions: compar- ison of drug-eluting stents, bare-metal stents and a mixed approach in patients at high and low risk of repeat revascularisation. EuroIntervention. 2010;5: 953–961. 20542781

[16] Stauffer J, Goy J, Duvoisin N, Radovanovic D, Rickli H, Erne P. Dramatic effect of early clopidogrel administration in reducing mortality and MACE rates in ACS pa- tients. Data from the Swiss registry AMIS-Plus. Swiss Med Wkly. 2012;142: w13573 10.4414/smw.2012.13573 22573491

[17] Engstrøm T, Kelbæk H, Helqvist S, Høfsten D, Kløvgaard L, Holmvang L, et al Complete revascularisation versus treatment of the culprit lesion only in patients with ST-segment elevation myocardial infarction and multivessel disease (DANAMI 3-PRIMULTI): an open-label, randomised controlled trial. Lancet. 2015;386: 665–671. 26347918 10.1016/s0140-6736(15)60648-1

[18] Gershlick AH, Khan JN, Kelly DJ, Greenwood JP, Sasikaran T, Curzen N, et al Randomized trial of complete versus lesion-only revascularization in patients undergoing primary percutaneous coronary intervention for STEMI and multivessel disease: the CvLPRIT trial. J Am Coll Cardiol. 2015;65: 963–972. 10.1016/j.jacc.2014.12.038 25766941 PMC4359051

[19] Wald DS, Morris JK, Wald NJ, Chase AJ, Edwards RJ, Hughes LO, et al Randomized trial of preventive angioplasty in myocardial infarction. N Engl J Med. 2013;369: 1115–1123. 10.1056/NEJMoa1305520 23991625

[20] Hlinomaz O. Multivessel coronary disease diagnosed at the time of primary PCI for STEMI: complete revascularization versus conservative strategy: the PRAGUE 13 trial. 2015. Available: http://sbhci.org.br/wp-content/uploads/2015/05/PRAGUE-13-Trial.pdf.

[21] Levine G, O'Gara P, Bates E, Blankenship J, Kushner F, Bailey S, et al. 2015 ACC/AHA/SCAI focused update on primary percutaneous coronary intervention for patients with ST-elevation myocardial infarction: an update of the 2011 ACCF/AHA/SCAI guideline for percutaneous coronary intervention and the 2013 ACCF/AHA guideline for the management of st-elevation myocardial infarction: a report of the American College of Cardiology/American Heart Association Task Force on Clinical Practice Guidelines and the Society for Cardiovascular Angiography and Interventions. J Am Coll Cardiol. 2015 Oct 21.

[22] Higgins J. Cochrane handbook for systematic reviews of interventions. Chichester, UK: Wiley; 2008.

[23] Liberati A, Altman D, Tetzlaff J, Mulrow C, Gøtzsche P, Ioannidis J, et al The PRISMA statement for reporting systematic reviews and meta-analyses of studies that evaluate healthcare interventions: explanation and elaboration. BMJ. 2009;339: b2700 10.1136/bmj.b2700 19622552 PMC2714672

[24] Wells G, Shea B, O'Connell D, Peterson J, Welch V, Losos M, et al. The Newcastle-Ottawa Scale (NOS) for assessing the quality if nonrandomized studies in meta-analyses. 2009. Available: http://www.ohri.ca/programs/clinical_epidemiology/oxford.htm.

[25] Politi L, Sgura F, Rossi R, Monopoli D, Guerri E, Leuzzi C, et al A randomised trial of target-vessel versus multi-vessel revascularisation in ST-elevation myocardial infarction: major adverse cardiac events during long-term follow-up Heart 2010;96: 662–667. 10.1136/hrt.2009.177162 19778920

[26] Di Mario C, Mara S, Flavio A, Imad S, Antonio M, Anna P, et al Single vs multi vessel treatment during primary angioplasty: results of the multicentre randomised HEpacoat for cuLPrit or multivessel stenting for Acute Myocardial Infarction (HELP AMI) Study. Int J Cardiovasc Intervent 2004;6: 128–133. 10.1080/14628840310030441 16146905

[27] Ochala A, Smolka GA, Wojakowski W, Dudek D, Dziewierz A, Krolikowski Z, et al The function of the left ventricle after complete multivessel one-stage percutaneous coronary intervention in patients with acute myocardial infarction. J Invasive Cardiol. 2004;16: 699–702. 15596873

[28] Tarasov R, Ganyukov V, Protopopov A, Barbarash O, Barbarash L. Six month results of randomized clinical trial: multivessel stenting versus staged revascularization for ST-elevation myocardial infarction patients with second generation drug eluting stents. Clin Med Res. 2014;3: 125–129.

[29] Corpus R, House J, Marso S, Grantham J, Huber K, Laster S, et al Multivessel percutaneous coronary intervention in patients with multivessel disease and acute myocardial infarction. Am Heart J. 2004;148: 493–500. 10.1016/j.ahj.2004.03.051 15389238

[30] Maamoun W, Elkhaeat N, Elarasy R. Safety and feasibility of complete simultaneous revascularization during primary PCI in patients with STEMI and multi-vessel disease. Egypt Heart J 2011;63: 39e43.

[31] Hannan E, Samadashvili Z, Walford G, Holmes D Jr, Jacobs A, Stamato N, et al Culprit vessel percutaneous coronary intervention versus multivessel and staged percutaneous coronary intervention for ST-segment elevation myocardial infarction patients with multivessel disease. JACC Cardiovasc Interv 2010;3: 22–31. 10.1016/j.jcin.2009.10.017 20129564

[32] Mohamad T, Bernal J, Kondur A, Hari P, Nelson K, Niraj A, et al Coronary revascularization strategy for ST elevation myocardial infarction with multivessel disease: experience and results at 1-year follow-up. Am J Ther 2011;18: 92–100. 10.1097/MJT.0b013e3181b809ee 20027110

[33] Varani E, Balducelli M, Aquilina M, Vecchi G, Hussien M, Frassineti V, et al Single or multivessel percutaneous coronary intervention in ST-elevation myocardial infarction patients. Catheter Cardiovasc Interv. 2008;72: 927–933. 10.1002/ccd.21722 18798239

[34] Kornowski R, Mehran R, Dangas G, Nikolsky E, Assali A, Claessen B, et al Prognostic impact of staged versus “one-time” multivessel percutaneous inter- vention in acute myocardial infarction: analysis from the HORIZONS-AMI (Harmonizing outcomes with revascularization and stents in acute myocardial infarction) trial. J Am Coll Cardiol. 2011;58: 704–711. 10.1016/j.jacc.2011.02.071 21816305

[35] Mauri L, Silbaugh TS, Garg P, Wolf RE, Zelevinsky K, Lovett A, et al Drug-eluting or bare-metal stents for acute myocardial infarction. N Engl J Med. 2008;359: 1330–1342. 10.1056/NEJMoa0801485 18815397

[36] Hannan EL, Racz MJ, McCallister BD, Ryan TJ, Arani DT, Isom OW, et al A comparison of three-year survival after coronary artery bypass graft surgery and percutaneous transluminal coronary angioplasty. J Am Coll Cardiol. 1999;33: 63–72. 9935010 10.1016/s0735-1097(98)00540-3

[37] Kastrati A, Dibra A, Spaulding C, Laarman G, Menichelli M, Valgimigli M, et al Meta-analysis of randomized trials on drug-eluting stents vs bare-metal stents in patients with acute myocardial infarction. Eur Heart J 2007;28: 2706–2713. 10.1093/eurheartj/ehm402 17901079

[38] De Luca G, Stone GW, Suryapranata H, Laarman GJ, Menichelli M, Kaiser C, et al Efficacy and safety of drug-eluting stents in ST-segment elevation myocardial infarction: a meta-analysis of randomized trials. Int J Cardiol. 2009;133: 213–222. 10.1016/j.ijcard.2007.12.040 18394731

[39] Smith SC Jr, Feldman TE, Hirshfeld JW Jr, Jacobs AK, Kern MJ, King S III, et al ACC/AHA/SCAI guideline update for percutaneous coronary intervention: a report of the American College of Cardiology/American Heart Association Task force on Practice Guidelines (ACC/AHA/SCAI Writing Committee to Update the 2001 Guidelines for Percutaneous Coronary Intervention). J Am Coll Cardiol Intv. 2006;47: e1–121. 10.1016/j.jacc.2005.12.001 16386656

[40] Goldstein JA, Demetriou D, Grines CL, Pica M, Shoukfeh M, O'Neill WW. Multiple complex coronary plaques in patients with acute myocardial infarction. N Engl J Med. 2000;343: 915–922. 10.1056/NEJM200009283431303 11006367

[41] Bell M, Gersh B, Schaff H, Holmes D Jr, Fisher L, Alderman E, et al Effect of completeness of revascularization on long-term outcome of patients with three-vessel disease undergoing coronary artery bypass surgery. A report from the Coronary Artery Surgery Study (CASS) Registry. Circulation. 1992;86: 446–457. 1638714 10.1161/01.cir.86.2.446

[42] Jones E, Weintraub W. The importance of completeness of revascularization during long-term follow-up after coronary artery operations. J Thorac Cardiovasc Surg 1996;112: 227–237. 8751484 10.1016/s0022-5223(96)70243-x

[43] Wu C, Dyer A, King S III, Walford G, Holmes D Jr, Stamato N, et al Impact of incomplete revascularization on long-term mortality after coronary stenting. Circ Cardiovasc Interv 2011;4: 413–421. 10.1161/CIRCINTERVENTIONS.111.963058 21972405 PMC3197764

[44] Bainey K, Mehta S, Lai T, Welsh R. Complete vs culprit-only revascularization for patients with multivessel disease undergoing primary percutaneous coronary intervention for ST-segment elevation myocardial infarction: a systematic review and meta-analysis. Am Heart J 2014;;167: 1–14.e12. 10.1016/j.ahj.2013.09.018 24332136

[45] Vlaar PJ, Mahmoud KD, Holmes DR Jr., van Valkenhoef G, Hillege HL, van der Horst IC, et al Culprit vessel only versus multivessel and staged percutaneous coronary intervention for multivessel disease in patients presenting with ST-segment elevation myocardial infarction: a pairwise and network meta-analysis. J Am Coll Cardiol. 2011;58: 692–703. 10.1016/j.jacc.2011.03.046 21816304

[46] Mager A, Vaknin Assa H, Lev E, Bental T, Assali A, Kornowski R. The ratio of contrast volume to glomerular filtration rate predicts outcomes after percutaneous coronary intervention for ST-segment elevation acute myocardial infarction. Catheter Cardiovasc Interv. 2011;78: 198–201. 10.1002/ccd.22828 20949583

[47] Hanratty C, Koyama Y, Rasmussen H, Nelson G, Hansen P, Ward M. Exaggeration of nonculprit stenosis during acute myocardial infarction: implication for immediate multivessel revascularization J Am Coll Cardiol. 2002;40: 911–916. 12225715 10.1016/s0735-1097(02)02049-1

[48] Dangas GD, George JC, Weintraub W, Popma JJ. Timing of staged percutaneous coronary intervention in multivessel coronary artery disease. JACC Cardiovasc Interv. 2010;3: 1096–1099. 10.1016/j.jcin.2010.09.005 20965476

[49] Barrett TD, Hennan JK, Marks RM, Lucchesi BR. C-reactive-protein-associated increase in myocardial infarct size after ischemia/reperfusion. J Pharmacol Exp Ther. 2002;303: 1007–1013. 10.1124/jpet.102.040600 12438521

[50] Marenzi G, Lauri G, Assanelli E, Campodonico J, De Metrio M, Marana I, et al Contrast-induced nephropathy in patients undergoing primary angioplasty for acute myocardial infarction. J Am Coll Cardiol Intv. 2004;44: 1780–1785. 10.1016/j.jacc.2004.07.043 15519007

[51] Glaser R, Selzer F, Faxon D, Laskey W, Cohen H, Slater J, et al Clinical progression of incidental, asymptomatic lesions discovered during culprit vessel coronary intervention. Circulation. 2005;111: 143–149. 10.1161/01.CIR.0000150335.01285.12 15623544

[52] Takano M, Inami S, Ishibashi F, Okamatsu K, Seimiya K, Ohba T, et al Angioscopic follow-up study of coronary ruptured plaques in nonculprit lesions J Am Coll Cardiol Intv. 2005;45: 652–658. 10.1016/j.jacc.2004.09.077 15734606

[53] Loh J, Kitabata H, Torguson R, Satler L, Kent K, Suddath W, et al Safety and feasibility of performing staged non-culprit vessel percutaneous coronary intervention within the index hospitalization in patients with ST-segment elevation myocardial infarction and multivessel disease. Cardiovasc Revasc Med. 2013;14 258–263. 10.1016/j.carrev.2013.05.005 24034862

