# Supplementary material for: Staged versus One-Time Complete Revascularization with Percutaneous Coronary Intervention in STEMI Patients with Multivessel Disease: A Systematic Review and Meta-Analysis
Source: PLoS One. 2017 Jan 20;12(1):e0169406. doi: 10.1371/journal.pone.0169406 (PMC5249143; doi:10.1371/journal.pone.0169406)
Supplement: S1 Table — (DOC) [file pone.0169406.s001.doc]

| **Section/topic** | **#** | **Checklist item** | **Reported on page #** |
| --- | --- | --- | --- |
| **TITLE** | | |  |
| Title | 1 | Staged Versus One-time Complete Revascularization With Percutaneous Coronary Intervention In STEMI patients with multivessel disease: A Systematic Review And Meta-analysis | 1 |
| **ABSTRACT** | | |  |
| Structured summary | 2 | Background: Patients with acute ST-elevation myocardial infarction (STEMI) are most commonly preferred to percutaneous coronary intervention (PCI). The 2015 ACC/AHA/SCAI guidelines recommend to consider multivessel PCI, either at the time of primary PCI or as staged procedure(Class IIb) modified from culprit vessel PCI (CV- PCI) lack of hemodynamic instability(Class III). However it is unknown which procedure between staged PCI (STAGED-PCI) and one-time complete PCI (MV-PCI) is more beneficial and safer to treat the nonculprit vessel during the primary PCI procedure. We performed a meta-analysis of all randomized and non-randomized controlled trials comparing STAGED-PCI with MV-PCI in patients with acute STEMI and MVD.  Methods : Studies about STEMI with multivessel disease receiving primary PCI in PUBMED,EMBASE and The Cochrane Register of Controlled Trials were searched from January 2004 to December 2014. The primary end point was long-term rates of major adverse cardiovascular events and its components, mortality, reinfarction, target-vessel revascularization. Data were combined using a fixed-effects model.  Results: Of 507 citations, 10 studies (4 randomized, 6 nonrandomized; 2060 patients, 1023 staged PCI and 1037 one-time complete multi-vessel PCI were included. STAGED-PCI compared to MV-PCI significantly reduced the mortality whenever in long-time (OR 0.44, 95% CI 0.29-0.66, P<0.0001, I2 = 0% and short-time (OR 0.23, 95% CI 0.1- 0.51, P = 0.0003, I2 = 0%). There was a trend toward reduced risk of MACE with Staged PCI compared with Multivessel PCI (OR 0.83, 0.62-1.12, P = 0.22, I2 = 0%), No difference between STAGED-PCI and MV-PCI was seen in reinfaction (OR 0.97, 0.61-1.55, P = 0.91, I2 = 0%),taget vessel revascularization (OR1.17, 95% CI 0.81-1.69, P = 0.40, I2 = 8%)  Conclusion: Staged strategy of nonculprit lesions improved short- and long-term survival,which should remain the standard approach undergoing primary PCI in patients with STEMI, as one-time complete multi-vessel PCI may be associated with a greater risk for mortality. However, more large randomized trials are required to confirm the optimal timing of a staged procedure to non-culprit vessel in STEMI. | 2 |
| **INTRODUCTION** | | |  |
| Rationale | 3 | Patients with acute ST-elevation myocardial infarction (STEMI) are most commonly preferred to percutaneous coronary intervention (PCI). The 2015 ACC/AHA/SCAI guidelines recommend to consider multivessel PCI, either at the time of primary PCI or as staged procedure(Class IIb) modified from culprit vessel PCI (CV- PCI) lack of hemodynamic instability(Class III). | 2 |
| Objectives | 4 | We performed a meta-analysis of all randomized and non-randomized controlled trials comparing STAGED-PCI with MV-PCI in patients with acute STEMI and MVD. | 2 |
| **METHODS** | | |  |
| Protocol and registration | 5 | Levine G, O'Gara P, Bates E, Blankenship J, Kushner F, Bailey S, et al. 2015 ACC/AHA/SCAI focused update on primary percutaneous coronary intervention for patients with ST-elevation myocardial infarction: an update of the 2011 ACCF/AHA/SCAI guideline for percutaneous coronary intervention and the 2013 ACCF/AHA guideline for the management of st-elevation myocardial infarction: a report of the American College of Cardiology/American Heart Association Task Force on Clinical Practice Guidelines and the Society for Cardiovascular Angiography and  Interventions. J Am Coll Cardiol. 2015 Oct 21. pii: : doi: 10.1016/j.jacc.2015.1010.1005. | 22 |
| Eligibility criteria | 6 | Clinical trials about STEMI with multivessel disease receiving primary PCI in English- language publications from January 2004 to December 2014.length of follow-up＞six months. English. | 5 |
| Information sources | 7 | PUBMED,EMBASE and The Cochrane Register of Controlled Trials. | 5 |
| Search | 8 | The following keywords and medical subject headings (MeSH) were used: ““coronary angioplasty,” “ST-elevation myocardial infarction,” “percutaneous coronary intervention,” “multivessel PCI,” “staged PCI,” “complete revascularization,” “non-culprit,” and “myocardial infarction.” | 5 |
| Study selection | 9 | Both RCTs and non-RCTs comparing staged vs multivessel PCI in patients with STEMI and MVD undergoing primary PCI without hemodynamic instability were included. | 5 |
| Data collection process | 10 | We screened the abstracts (i.e., unpublished citations) and full-text citations for eligibility in the meta-analysis. To eliminate negative publication bias, unpublished citations were also included, and the relevant references were collected through a manual search. | 5 |
| Data items | 11 | Data were extracted systematically from the intervention (S-PCI) and control (MV-PCI). The primary end points were short-term mortality,long-term rates of major adverse cardiovascular events and their components—mortality, reinfarction, and target-vessel revascularization.. | 6 |
| Risk of bias in individual studies | 12 | The Cochrane Collaboration tool was used to assess the quality of the abstracted studies to assess the risk of bias in the RCTs. The Newcastle Ottawa Scale was used to evaluate the quality of the non-RCTs. | 5 |
| Summary measures | 13 | All statistical analyses were performed using Review Manager (RevMan 5.2, Cochrane Collaboration, Nordic Cochrane Center, Copenhagen, Denmark). Odds ratios (ORs) with 95% CIs were used as summary estimates. Given the low event rates and small size of selected studies, the Mantel-Haenszel method was used to calculate the pooled OR with the fixed-effects model. | 6 |
| Synthesis of results | 14 | Study heterogeneity was measured using the I^2^ index and Cochran’s Q, where an I^2^ greater than 60 and P<0.1 represent severe heterogeneity. | 6 |

Page 1 of 2

| **Section/topic** | **#** | **Checklist item** | **Reported on page #** |
| --- | --- | --- | --- |
| Risk of bias across studies | 15 | To eliminate negative publication bias, unpublished citations were also included, and the relevant references were collected through a manual search. Given the low event rates and small size of selected studies, the Mantel-Haenszel method was used to calculate the pooled OR with the fixed-effects model. A “funnel plot” approach was used to avoid the potential for publication bias. | 6 |
| Additional analyses | 16 | Sensitivity analyses were performed to explore heterogeneity. Depending on the study design, a subgroup of the RCTs and non-RCTs was generated for each outcome to help explain heterogeneity. | 6 |
| **RESULTS** | | |  |
| Study selection | 17 | As shown in Figure 1, 850 abstracts were retrieved, and 40 were selected. Of these 40 eligible full-text studies, 30 studies were excluded due to lack of STEMI (n=9), lack of multivessel revascularization (n=5), failure to identify the control group (n=4), cardiogenic shock (n=5), and inclusion of coronary artery bypass grafting surgery (n=7). Ten studies fulfilled the eligibility criteria and were included in the present systematic review. |  |
| Study characteristics | 18 | 1. Politi ,214 patients, In-hospital mortality; long-term mortality, cardiac death, MI, repeat revascularization, rehospitalization, CABG, PCI, MACE,   Politi L, Sgura F, Rossi R, Monopoli D, Guerri E, Leuzzi C, et al. A randomised trial of  target-vessel versus multi-vessel revascularisation in ST-elevation myocardial infarction:  major adverse cardiac events during long-term follow-up Heart 2010;96: 662-667.   1. Horizon, 668 patients, 1-y MACE   Kornowski R, Mehran R, Dangas G, Nikolsky E, Assali A, Claessen B, et al. Prognostic impact of staged versus “one-time” multivessel percutaneous inter- vention in acute myocardial infarction: analysis from the HORIZONS-AMI (Harmonizing outcomes with revascularization and stents in acute myocardial infarction) trial. . J Am Coll Cardiol. 2011;58: 704-711.   1. Ochala, 92 patients, LVEF, all causes of death, AMI, urgent revascularization (including TVR), major and minor bleeding complications, worsening of the CCS class, unstable angina, cardiovascular hospitalization   Ochala A, Smolka GA, Wojakowski W, Dudek D, Dziewierz A, Krolikowski Z, et al. The  function of the left ventricle after complete multivessel one-stage percutaneous coronary intervention in patients with acute myocardial infarction. J Invasive Cardiol. 2004;16: 699-702.   1. Tarasov，89 patients, 6 month MACE   Tarasov R, Ganyukov V, Protopopov A, Barbarash O, Barbarash L. Six month results of randomized clinical trial: multivessel stenting versus staged revascularization for ST-elevation myocardial infarction patients with second generation drug eluting stents. Clin Med Res. 2014;3: 125-129.   1. Corpus, 506 patients, In-hospital mortality; 30-d mortality, reinfarction, TVR, CABG, MACE; 1-y mortality, reinfarction, TVR, CABG, MACE   Corpus R, House J, Marso S, Grantham J, Huber K, Laster S, et al. Multivessel percutaneous coronary intervention in patients with multivessel disease and acute myocardial infarction. Am Heart J. 2004;148: 493-500.   1. Khattab,70 patients, 30-d mortality, MI, TVR, stent thrombosis, CVA, bleeding, MACE; 1-y mortality, MI, TVR, non-TVR, total revascularizations, MACE   Khattab A, Abdel-Wahab M, Röther C, Liska B, Toelg R, Kassner G, et al. Multi-vessel stenting during primary percutaneous coronary intervention for acute myocardial infarction. A single-center experience Clin Res Cardiol. 2008;97: 32–38.   1. Hannan, 1434 patients, In-hospital mortality; 12-mo mortality; 24-mo mortality; 42-mo mortality   Hannan E, Samadashvili Z, Walford G, Holmes Jr D, Jacobs A, Stamato N, et al. Culprit vessel percutaneous coronary intervention versus multivessel and staged percutaneous coronary intervention for ST-segment elevation myocardial infarction patients with multivessel disease. JACC Cardiovasc Interv 2010;3: 22-31.   1. Mohamad, 63 patients, 1-y mortality, MACE   Mohamad T, Bernal J, Kondur A, Hari P, Nelson K, Niraj A, et al. Coronary revascularization strategy for ST elevation myocardial infarction with multivessel disease: experience and results at 1-year follow-up. Am J Ther 2011;18: 92-100.   1. Varani, 399 patients, In-hospital mortality, PCI, major vascular complications; 30-d mortality; long-term (630±366 d) mortality   Varani E, Balducelli M, Aquilina M, Vecchi G, Hussien M, Frassineti V, et al. Single or multivessel percutaneous coronary intervention in ST-elevation myocardial infarction patients. Catheter Cardiovasc Interv. 2008;72: 927-933.   1. Maamoun, 19 patients,1-y mortality, MACE   Maamoun W, Elkhaeat N, Elarasy R. Safety and feasibility of complete simultaneous revascularization during primary PCI in patients with STEMI and multi-vessel disease. Egypt Heart J 2011;63: 39e43. | 7-11 |
| Risk of bias within studies | 19 | The risk of bias for each included randomized study were estimated by the Cochrane Collaboration Tool, All of the 4 RCT have adequate sequence generation, allocation concealment, Incompleted data addressed, free of selective reporting,free of other bias.  The high quality of the non-RCTs was indicated by the Newcastle Ottawa Scale score of ≥6/8.  The funnel plot revealed no publication bias. | 13 |
| Results of individual studies | 20 | long-term MACE short-term mortality long-term mortality long-term Re-mi long-term TVR (OR CI)  horizon 0.67{0.44,1.03} 0.44{0.14,1.39} 0.69{0.35,1.34} 0.93{0.53,1.61}  politi 0.83{0.36,1,93} 0.19{0.01,4.12} 0.64{0.17,2.40} 2.07{0.36,11.69} 1.38{0.45,4.23}  ochala 1.43{0.54,3.73} 1.50{0.32,7.11} 1.12{0.43,2.92}  tarasov 3.28{0.13,82.77} 0.14{0.01,2.85} 0.20{0.01,4.39}  corpus 1.37{0.57,3.31} 0.1{0.02,0.46} 0.44{0.14,1.39} 4.44{0.57,34.74} 9.62{1.25,73.69}  Khattab 1.15{0.37,3.57} 1.26{0.11,14.53} 0.82{0.13,5.28} 1.00{0.29,3.39} 1.15{0.37,3.57}  Hannan 0.34{0.10,1.15} 0.52{0.25,1.07}  Mohamad 0.95{0.14,6.28} 0.50{0.05,4.67}  Varani 0.23{0.02,0.93}  Maamoun 0.68{0.23,1.99} 1.17{0.07,19.42} 0.75{0.19,2.90} | 30-32 |
| Synthesis of results | 21 | There was a trend toward reduced risk of MACE with S-PCI compared with MV-PCI with no heterogeneity, although the trend did not reach statistical significance (OR 0.83, 95% CI 0.62–1.12, P=0.22, I2=0%),  Mortality was significantly lower for S-PCI compared with MV-PCI. Improved in-hospital survival was observed for S-PCI, with no heterogeneity (OR 0.23, 95% CI 0.1–0.51, P=0.0003, I2=0%).  The combined analysis indicated a survival benefit for S-PCI compared with MV-PCI (OR 0.44, 95% CI 0.29–0.66, P<0.0001, I2=0%).  There was no significant difference between S-PCI and MV-PCI. S-PCI had no effect on re-MI, with no heterogeneity (OR 0.97, 95% CI 0.61–1.55, P=0.91, I2=0%).  Comparing S-PCI with MV-PCI revealed no significant difference in target revascularization in the RCTs [25,27,34], with no heterogeneity (OR 0.98, 95% CI 0.64–1.51, P=0.09, I2=0%) (Figure 7). In the non-RCTs [10,29,30], enhanced revascularization was observed for MV-PCI, with moderate heterogeneity (OR 1.84, 95% CI 0.90–3.79, P=0.10, I2=59%), mainly driven by the results of the non-RCT performed by Corpus. | 11-13 |
| Risk of bias across studies | 22 | Comparing S-PCI with MV-PCI revealed no significant difference in target revascularization in the RCTs [25,27,34], with no heterogeneity (OR 0.98, 95% CI 0.64–1.51, P=0.09, I2=0%) (Figure 7). In the non-RCTs [10,29,30], enhanced revascularization was observed for MV-PCI, with moderate heterogeneity (OR 1.84, 95% CI 0.90–3.79, P=0.10, I2=59%), mainly driven by the results of the non-RCT performed by Corpus. | 13 |
| Additional analysis | 23 | no |  |
| **DISCUSSION** | | |  |
| Summary of evidence | 24 | The risk of bias for each included randomized study were estimated by the Cochrane Collaboration Tool, All of the 4 RCT have adequate sequence generation, allocation concealment, Incompleted data addressed, free of selective reporting,free of other bias.  The high quality of the non-RCTs was indicated by the Newcastle Ottawa Scale score of ≥6/8.  The funnel plot revealed no publication bias.  Our meta-analysis provides new insights on the efficacy and safety of staged PCI compared with one-time complete PCI in patients with STEMI. | 13,18 |
| Limitations | 25 | In our meta-analysis, we were forced to include observational non-randomized studies due to a lack of randomized data. We performed randomized vs nonrandomized stratified analyses for the pooled estimate. However, many selection biases and confounding factors remained in the observational studies, even after statistical adjustment. Unpublished abstracts were also included to reduce publication bias. The data of the original included studies were limited to analysis at the trial level rather than the patient level. Therefore, we could not adjust the baseline characteristics of the included patients and multivariate factors; the follow-up and admission medications were also not captured. Moreover, the impact of chronic total occlusions was not fully evaluated due to the absence of reports in most selected studies. A staged and planned strategy for non-culprit vessel PCI may be preferable for these patients given the risk and difficulties in attempting a chronic total occlusion. In addition, when patients wait for staged or planned PCI, clinical events may occur; these events were not adequately described in the included studies. Finally, survival selection bias in staged PCI patients can potentially affect long-term survival. Therefore, we excluded patients in cardiogenic shock and performed a sensitivity analysis of all studies, which confirmed the survival benefit. | 17,18 |
| Conclusions | 26 | We observed reduced short- and long-term mortality with a strategy of staged PCI. The results of our study suggest that PCI of the non-culprit vessel should be staged and that many factors and conditions influence the decision of when to stage PCI. However, our findings require additional large-scale, multicenter, randomized controlled studies for confirmation. | 18 |
| **FUNDING** | | |  |
| Funding | 27 | no |  |

*From:*  Moher D, Liberati A, Tetzlaff J, Altman DG, The PRISMA Group (2009). Preferred Reporting Items for Systematic Reviews and Meta-Analyses: The PRISMA Statement. PLoS Med 6(7): e1000097. doi:10.1371/journal.pmed1000097

For more information, visit: **www.prisma-statement.org**.

Page 2 of 2
