# Supplementary material for: Staged versus One-Time Complete Revascularization with Percutaneous Coronary Intervention in STEMI Patients with Multivessel Disease: A Systematic Review and Meta-Analysis
Source: PLoS One. 2017 Jan 20;12(1):e0169406. doi: 10.1371/journal.pone.0169406 (PMC5249143; doi:10.1371/journal.pone.0169406)
Supplement: S1 File — (DOCX) [file pone.0169406.s002.docx]

**Original data of the study**

**Table A. Date of long-term MACE**

|  | Staged pci | | Multivessal pci | |
| --- | --- | --- | --- | --- |
| study | events | total | events | total |
| Horizon 2011 | 51 | 393 | 50 | 275 |
| Ochala 2004 | 12 | 44 | 10 | 48 |
| Politi 2010 | 13 | 65 | 15 | 65 |
| Tarasov 2014 | 1 | 43 | 3 | 46 |
| Corpus 2004 | 53 | 126 | 9 | 26 |
| Khattab 2007 | 12 | 45 | 6 | 25 |
| Maamoun 2011 | 7 | 36 | 11 | 42 |
| Mohamad 2011 | 5 | 12 | 3 | 7 |

**Table B. Date of short-term mortality**

|  | Staged pci | | Multivessal pci | |
| --- | --- | --- | --- | --- |
| study | events | total | events | total |
| Ochala 2004 | 0 | 44 | 0 | 48 |
| Politi 2010 | 0 | 65 | 2 | 65 |
| Corpus 2004 | 3 | 126 | 5 | 26 |
| Hannan 2009 | 3 | 259 | 17 | 503 |
| Khattab 2007 | 2 | 45 | 1 | 28 |
| Varani 2008 | 1 | 96 | 12 | 147 |

**Table C. Date of long-term mortality**

|  | Staged pci | | Multivessal pci | |
| --- | --- | --- | --- | --- |
| study | events | total | events | total |
| Horizon 2011 | 9 | 393 | 25 | 275 |
| Ochala 2004 | 0 | 44 | 0 | 48 |
| Politi 2010 | 4 | 65 | 6 | 65 |
| Tarasov 2014 | 1 | 43 | 0 | 46 |
| Corpus 2004 | 12 | 126 | 5 | 26 |
| Khattab 2007 | 3 | 45 | 2 | 25 |
| Maamoun 2011 | 1 | 36 | 2 | 42 |
| Mohamad 2011 | 2 | 12 | 2 | 7 |
| Hannan 2009 | 10 | 259 | 36 | 503 |

**Table D. Date of long-term Re-mi**

|  | Staged pci | | Multivessal pci | |
| --- | --- | --- | --- | --- |
| study | Events | total | events | total |
| Horizon 2011 | 18 | 393 | 18 | 275 |
| Ochala 2004 | 4 | 44 | 3 | 48 |
| Politi 2010 | 4 | 65 | 2 | 65 |
| Tarasov 2014 | 0 | 43 | 3 | 46 |
| Corpus 2004 | 19 | 126 | 1 | 26 |
| Khattab 2007 | 9 | 45 | 5 | 25 |
| Maamoun 2011 | 1 | 36 | 1 | 42 |

**Table E. Date of long-term TVR**

|  | Staged pci | | Multivessal pci | |
| --- | --- | --- | --- | --- |
| study | events | total | events | total |
| Horizon 2011 | 32 | 393 | 24 | 275 |
| Ochala 2004 | 11 | 44 | 11 | 48 |
| Politi 2010 | 8 | 65 | 6 | 65 |
| Tarasov 2014 | 0 | 43 | 2 | 46 |
| Corpus 2004 | 35 | 126 | 1 | 26 |
| Khattab 2007 | 12 | 45 | 6 | 25 |
| Maamoun 2011 | 4 | 36 | 6 | 42 |
